# Eyelid Spindle Cell Lipoma: Case Report and Review of Three Patients in Literature

**DOI:** 10.1002/ccr3.70097

**Published:** 2025-01-07

**Authors:** Toshihiko Matsuo, Kiyoshi Yamada, Yasumasa Monobe, Takehiro Tanaka

**Affiliations:** ^1^ Graduate School of Interdisciplinary Science and Engineering in Health Systems Okayama University Okayama Japan; ^2^ Department of Ophthalmology Okayama University Hospital Okayama Japan; ^3^ Plastic and Reconstructive Surgery Kousei Hospital Okayama Japan; ^4^ Department of Pathology General Medical Center, Kawasaki Medical School Okayama Japan; ^5^ Okayama Medical Laboratories Inc. Kurashiki Japan; ^6^ Department of Pathology, Graduate School of Medicine, Dentistry, and Pharmaceutical Sciences Okayama University Okayama Japan

**Keywords:** CD34, eyelid, orbital bony edge, pathology, spindle cell lipoma

## Abstract

A 39‐year‐old woman presented a saucer‐shaped mass in the left upper eyelid and underwent the extirpation at local anesthesia. Pathologically, collagen fibers, capillaries, small vessels, and CD34‐positive spindle cells were dispersed among mature adipose tissues, indicative of spindle cell lipoma. Long‐lasting cyst‐like eyelid masses would be usually dermoid cysts, and spindle cell lipoma would be listed as a rare pathological diagnosis in differential diagnoses of cyst‐like lesions in the upper and lower eyelid.

## Graphical abstract

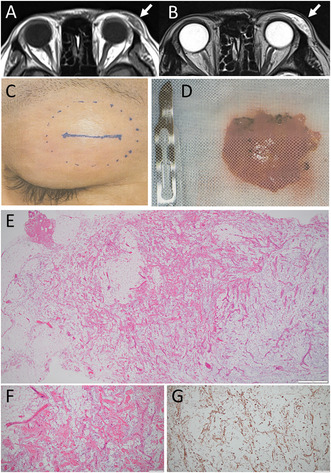

## 1 Introduction

Lipoma is a benign tumor primarily of mature adipose tissues that are intermingled with different mesenchymal cells such as smooth muscle cells, vascular cells, and other premature cells called spindle cells [1, 2, 3]. The common location of lipoma is the subcutaneous tissues of the back and neck. Pathologically, lipoma is designated differentially as spindle cell lipoma, pleomorphic lipoma, angiolipoma, myolipoma, and fibrolipoma, based on the presence of mesenchymal cell types. Among these different subclasses of lipomas, spindle cell lipoma is basically rare from the viewpoint of whole body. Orbital spindle cell lipoma has been sparsely described as case reports [4, 5, 6, 7, 8, 9, 10, 11], and eyelid spindle cell lipoma has been reported only in three patients up to date [12, 13, 14]. In this study, we presented a patient who showed a preseptal eyelid saucer‐shaped mass with the clinical diagnosis of a dermoid cyst. The resection of the mass revealed spindle cell lipoma by the pathological examination. We also reviewed three patients with eyelid spindle cell lipoma in the literature [12, 13, 14].

## 2 Case Presentation

**FIGURE 1:**
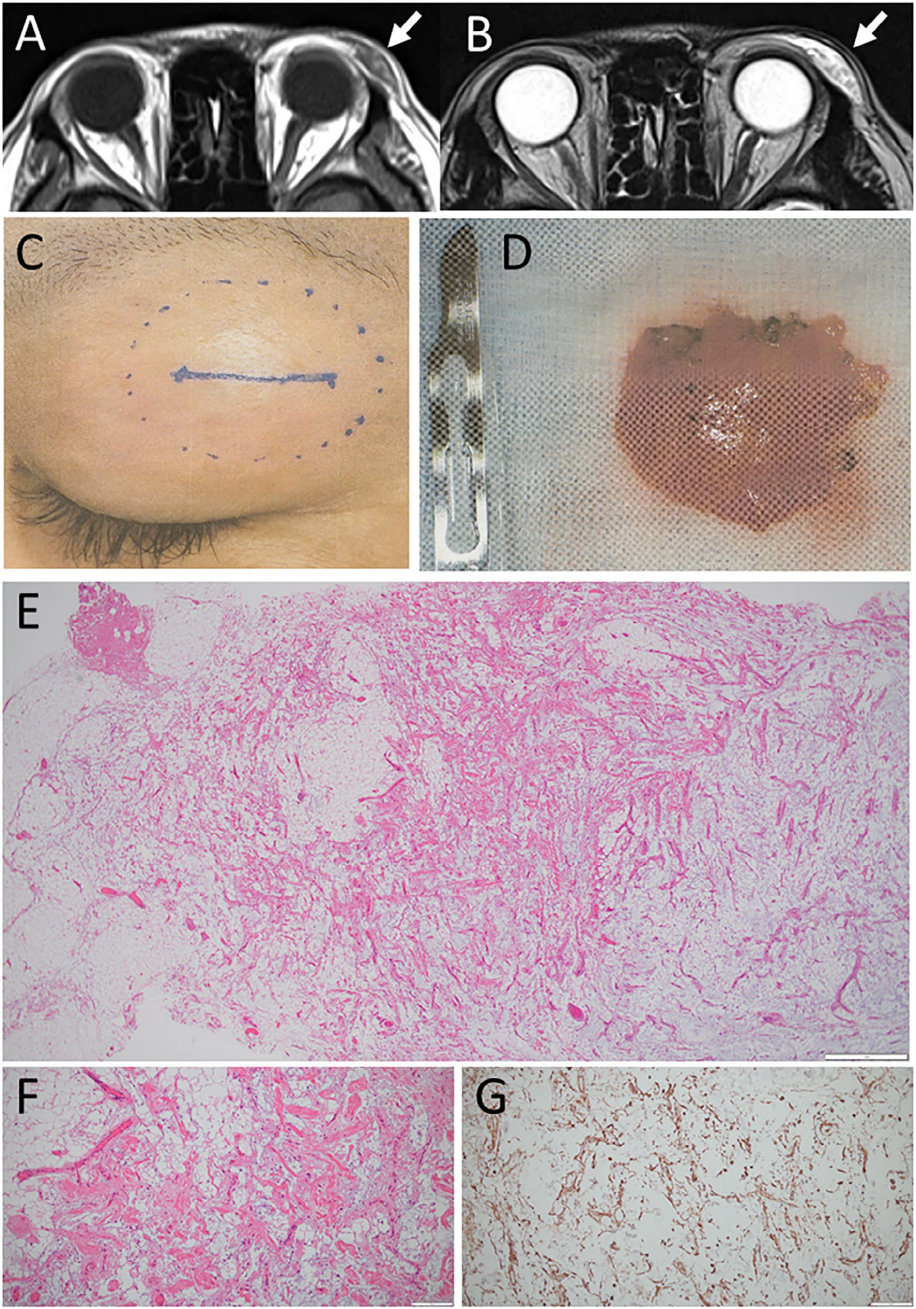
Left upper eyelid saucer‐shaped mass with inhomogeneous content on T1‐weighted axial image (arrow, A) and T2‐weighted axial image (arrow, B) of magnetic resonance imaging. Left upper eyelid subcutaneous flat mass with 2 × 2.5 cm in size at surgery (C) and macroscopic view of the extirpated mass with size reference of no. 15 curved blade (D). Note incision line and area of the mass are marked on the skin at surgery by local anesthesia (C). Collagen fibers, capillaries and small vessels, and spindle cells in small number which are dispersed among mature adipose tissues with varying sizes (E in low magnification, F in high magnification). Spindle cells are positive for CD34, supporting the diagnosis of spindle cell lipoma. Bar = 1000 μm in E, 200 μm in (F and G).

A 39‐year‐old woman experienced recent enlargement of the left upper eyelid mass on the temporal side which she had noticed from the age of about 12 years. The mass was saucer‐shaped and elastic‐hard, about 2‐cm‐diameter in size, and appeared to have adhesion to the supero‐temporal orbital bony edge. She was healthy with no medication and had no past history. The physical examinations showed nothing to be noted. Complete blood cell counts, blood chemistry, blood glucose, serological tests for syphilis, and urinalysis were all within normal limits. Magnetic resonance imaging (Figure 1A,B) showed a well‐defined, saucer‐shaped mass with inhomogeneous content of mixed lipid and water in the left upper eyelid on the temporal side with probable adhesion to the orbital bony edge.

## 3 Surgery and Pathology

With the clinical diagnosis of a dermoid cyst, the patient underwent the extirpation at local anesthesia (Figure 1C). The saucer‐shaped mass (Figure 1D) was located anterior to the orbital septum and had firm adhesion to the supero‐temporal orbital bony edge. The pathological examination of the mass showed that collagen fibers, capillaries and small vessels, and spindle cells in small number were dispersed among mature adipose tissues with different sizes (Figure 1E,F). The spindle cells were positive for CD34 (Figure 1G), indicative of spindle cell lipoma. The patient had no complication and was well after the surgery.

## 4 Discussion

To analyze similar cases, PubMed and Google Scholar were searched with key words, “eyelid” and “spindle cell lipoma”. A sufficient description was found in three patients (Table 1) [12, 13, 14]. One patient with pathological diagnosis of simple lipoma [15] was not included in this review. The four patients, including the present patient (Case 4), were 3 women and one man with the age at the surgery varying from 39 to 82 years. Most patients noticed the eyelid mass in the long period of time and experienced the recent painless enlargement. The laterality was on the left side in three patients and on the right side in one (Case 3). Two patients showed the masses in the upper eyelid and the other 2 showed on the lower eyelid. The location of masses in the eyelid was deviated to the temporal side in the present patient (Case 4) or to the nasal side (Case 2) or was located in the center (Cases 1 and 3). Computed tomography (CT) scans in two patients (Cases 1 and 3) showed well‐defined cyst‐like appearance with low density whereas magnetic resonance imaging in the present patient (Case 4) also showed well‐defined cyst‐like appearance with mixed content of lipid and water.

**TABLE 1:** Clinical features of four patients with preseptal eyelid spindle cell lipoma, including the present patient.

Case no. /Gender	Age at surgery	Symptoms	Duration	Location	Imaging	Periosteal adhesion at surgery	Pathological diagnosis	Authors (year)
1/Female	82 years	3‐week enlargement	For a few years	Left upper eyelid at center	Low‐density cyst‐like appearance on CT	No	Spindle cell lipoma	Mawn, Jordan, and Olberg [12]
2/Female	67 years	No recent change	For 50 years	Left lower eyelid on nasal side	No description	Yes	Myolipoma Spindle cells positive for SMA and desmin	Sharara, Lee, and Weir [13]
3/Male	47 years	Painless 1‐year enlargement	For a year	Right lower eyelid at center	Low‐density cyst‐like appearance on CT	Not described	Spindle cell lipoma Spindle cells positive for CD34	Charles and Belinsky [14]
4/Female	39 years	Recent enlargement	From 12 years old	Left upper eyelid on temporal side	Saucer‐shaped cyst‐like appearance with mixed lipid and water content on MRI	Yes	Spindle cell lipoma Spindle cells positive for CD34	This case

Abbreviations: CT, computed tomography scan; MRI, magnetic resonance imaging; SMA, smooth muscle actin.

At surgical extirpation, all masses in the four patients were located at preseptal area of the eyelid with periosteal adhesion at one point in two patients who showed a mass deviated to the nasal side (Case 2) or the temporal side (Case 4). The pathological diagnosis was spindle cell lipoma in three patients of which two were reported to show CD34‐positive spindle cells (Cases 3 and 4). One patient (Case 2) was diagnosed as myolipoma, based on spindle cells positive for smooth muscle actin.

The eyelid dermoid cyst is ranked at the top in the list of differential diagnoses [16]. The differential points would be (1) dermoid cysts are more spherical or ovoid in shape, (2) the dermoid content is more homogeneous than spindle cell lipoma, and (3) dermoid cysts are detected earlier in life. The eyelid dermoid cysts are frequently adherent to the temporal orbital bony edge. Under the circumstances, the periosteal adhesion would be a common sign between the dermoid cyst and spindle cell lipoma. In conclusion, spindle cell lipoma would be listed in differential diagnoses of cyst‐like lesions in the upper and lower eyelid.

## Author Contributions

**Toshihiko Matsuo:** conceptualization, data curation, formal analysis, investigation, methodology, project administration, resources, validation, visualization, writing – original draft. **Kiyoshi Yamada:** data curation, formal analysis, resources, validation, visualization, writing – review and editing. **Yasumasa Monobe:** investigation, resources, visualization. **Takehiro Tanaka:** data curation, formal analysis, investigation, methodology, resources, visualization, writing – review and editing.

## Ethics Statement

Ethics committee review was not applicable due to the case report design, based on the Ethical Guidelines for Medical and Health Research Involving Human Subjects, issued by the Government of Japan.

## Consent

Written consent was obtained from the patient for her anonymized information to be published in this article.

## Conflicts of Interest

The authors declare no conflicts of interest.

## Data Availability

Additional data are available upon reasonable request to the corresponding author.
